# Detection of Vegetable Oil Adulteration in Pre-Grated Bovine Hard Cheeses via ^1^H NMR Spectroscopy

**DOI:** 10.3390/molecules28030920

**Published:** 2023-01-17

**Authors:** Colleen L. Ray, James A. Gawenis, Madison P. Bylo, Jonny Pescaglia, C. Michael Greenlief

**Affiliations:** 1 Department of Chemistry, University of Missouri, 601 S. College Avenue, Columbia, MO 65211, USA; 2 Sweetwater Science Laboratories, Glasgow, MO 65264, USA

**Keywords:** NMR, bovine cheese, food adulteration, ratiometrics

## Abstract

Adulteration of food products is a widespread problem of great concern to society and dairy products are no exception to this. Due to new methods of adulteration being devised in order to circumvent existing detection methods, new detection methods must be developed to counter fraud. Bovine hard cheeses such as Asiago, Parmesan, and Romano are widely sold and consumed in pre-grated form for convenience. Due to being processed products, there is ample opportunity for the introduction of inexpensive adulterants and as such, there is concern regarding the authenticity of these products. An analytical method was developed using a simple organic extraction to verify the authenticity of bovine hard cheese products by examining the lipid profile of these cheeses via proton Nuclear Magnetic Resonance (NMR) spectroscopy. In this study, 52 samples of pre-grated hard cheese were analyzed as a market survey and a significant number of these samples were found to be adulterated with vegetable oils. This method is well suited to high throughput analysis of these products and relies on ratiometrics of the lipids in the samples themselves. Genuine cheeses were found to have a very consistent lipid profile from sample to sample, improving the power of this approach to detect vegetable oil adulteration. The method is purely ratiometric with no need for internal or external references, reducing sample preparation time and reducing the potential for the introduction of error.

## 1. Introduction

Bovine hard cheeses are a widely consumed dairy product throughout much of the world and pre-grated products made from these cheeses are popular as a condiment for many foods. The majority of these products are composed of grated cheese with small amounts of antimycotic preservatives such as potassium sorbate, which was used in many samples in this study. Anti-caking agents such as cellulose powder are also used in most samples tested. Some grated cheeses with no additives are also encountered. Adulteration of these cheese products has previously been discovered involving the addition of cellulose powder as a filler at levels far beyond those sufficient to prevent caking [1]. Food adulteration leads to a product that is cheaper and is sold as the original product. This results in consumers buying a product that is not what they expected and is often inferior to the unadulterated version. Due to the nature of this product’s manufacture and its typical use as a garnish on other foods, it would seem a prime candidate for adulteration with a low chance of discovery by the consumer.

NMR analysis of food products is a powerful tool for the detection of adulteration. It is ideal for analyses of this type due to high sample throughput, the ability to discriminate based on structural differences of metabolites with similar masses, and the ability to examine samples in either their native state or with little sample preparation. Despite these abilities, NMR does not appear to have previously been applied to detecting the adulteration of cheese with vegetable oils.

NMR simplifies the analysis of structural features in analytes, and this becomes a very powerful tool to detect food fraud. While structural information can be inferred from chromatography retention times or tandem mass spectrometry, it is inherent to NMR experiments. It is particularly valuable when identifying lipids in edible oils or cheese. For example, both α-linolenic acid and γ-linolenic acid are 18-carbon, triply unsaturated fatty acids found in edible oils with identical molecular weights of 278.436 Da. Via LC-MS these fatty acids would only vary by retention time assuming adequate separation in the chromatography step. Tandem mass spectrometry can also be used to differentiate these, but this involves a separate experiment. However, as α-linolenic acid is an ω-3 fatty acid with an unsaturated bond located three carbons from the terminal methyl group, its methyl group displays a distinct triplet proton resonance at approximately 0.97 ppm, where the ω-6 γ-linolenic’s methyl signal is closer to 0.85 ppm [2]. This allows for much easier quantification of ω-3 fatty acids with NMR and speeds the determination of a sample’s lipid profile. Additionally, this structural information is invaluable when comparing levels of polyunsaturated fatty acids, total numbers of unsaturated bonds, and ω-3 fatty acid levels present in a sample. While not an exhaustive analysis of the sample’s composition, it is often sufficient to determine the authenticity of said sample.

The simplicity of NMR sample preparation is a natural complement to its inherent speed of analysis. Simple dilution or liquid extractions are much less labor intensive than some sample preparation regimens required for other methods. Significant improvements in laboratory throughput can be achieved by eliminating the need for steps such as derivatization for gas chromatography of non-volatile compounds, hydrolysis of triacylglycerols for analysis of lipids in mass spectrometry, and with the additional benefit of reducing the opportunity for sample contamination or errors. This also has the benefit of analyzing samples in their native or near-native state without chemical modification.

Analysis of cheese via NMR has been performed for quite some time. However, previous works were typically focused on aqueous extracts in order to determine point of origin [3,4], or a combination of origination and the process of cheese ripening [5]. ^1^H NMR-based lipid profiling of cheese has been performed before, however, this approach involved a lengthy soxhlet extraction step making it less appealing for routine screening of samples, and the study did not address adulteration [5]. The spectral differences between the lipid profiles of genuine cheese and imitation cheese prepared from vegetable oil was demonstrated previously but this method was also not applied to the detection of adulterated products in a market survey [6]. Mass spectrometry has also been used to profile the lipids in cheese but was similarly not used as an approach for detecting adulteration [7].

The aim of this study was to create and test a method for the analysis of hard cheese products with the aim of detecting vegetable oil adulterants. The method was designed to be rapid in order to facilitate its use in high-throughput situations. The difference in lipid profiles between cheese and vegetable oils makes the detection of adulterated cheeses relatively straightforward with a simple ratiometric analysis.

## 2. Results and Discussion

**Figure 1:**
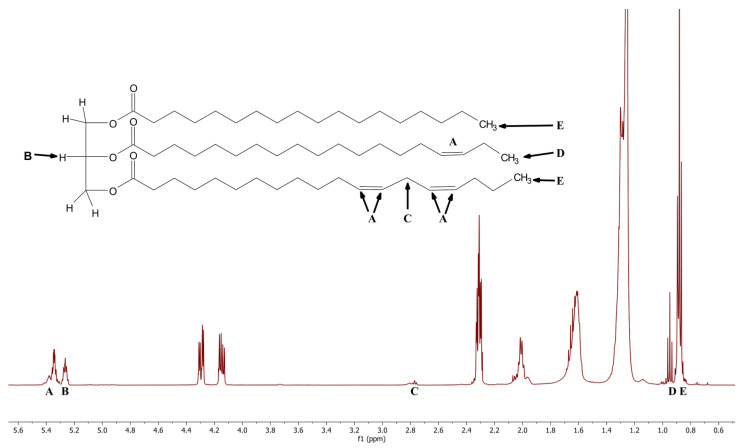
500 MHz ^1^H NMR spectrum of sample B1 (see Table 4 for additional information) with a model triacylglycerol diagram of peaks used for analysis. The A through E labels on the chemical structure correspond to the protons that give rise to the labeled resonances in the NMR spectrum.

A number of ungrated bovine hard cheeses were obtained. A description of the cheeses can be found in the Section 3 and are designated as “baseline” samples due to being representative of genuine cheese. ^1^H NMR spectra were obtained for each of these cheese samples. The samples were found to have a spectrum consistent with lipids in the form of triacylglycerols. Figure 1 is the ^1^H NMR spectrum of one the parmesan baseline cheeses. The labels A through E below the spectrum indicate the region of interest for this study. The chemical structure in Figure 1 is an idealized triacylglycerol. The five labels, A through E, on the structure indicate the protons that give rise to the resonances obtained in the spectrum.

The five regions of interest were examined using the raw integral values from the associated peaks. (Table 1 and Figure 1). Several different integral ratios were determined for the baseline cheeses. The results of this ratio comparison are presented in Table 2. The baseline cheeses showed acceptable consistency in all four ratios despite the variety of cheeses.

**Figure 2:**
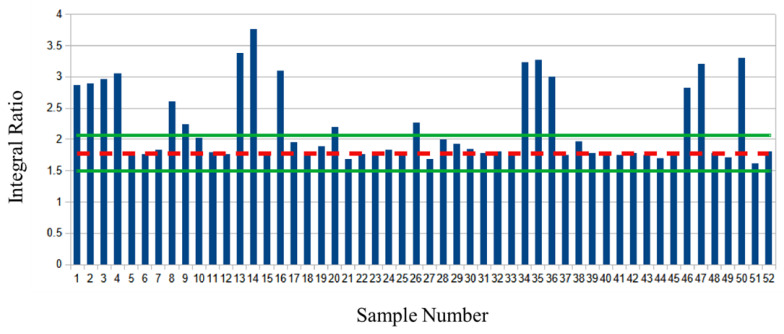
Unsaturated bond to glycerol C2 proton ratio of market survey samples. The mean ratio of baseline samples is depicted with a dashed red line, green lines are the mean plus or minus two standard deviations.

**Figure 3:**
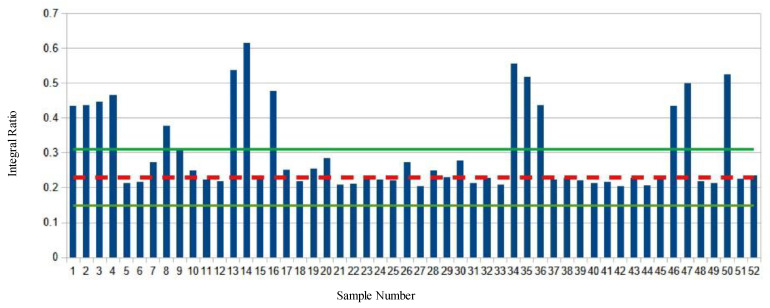
Polyunsaturated protons to glycerol C2 proton ratio of market survey samples. The mean ratio of baseline samples is depicted with a dashed red line, green lines are the mean plus or minus two standard deviations.

**Figure 4:**
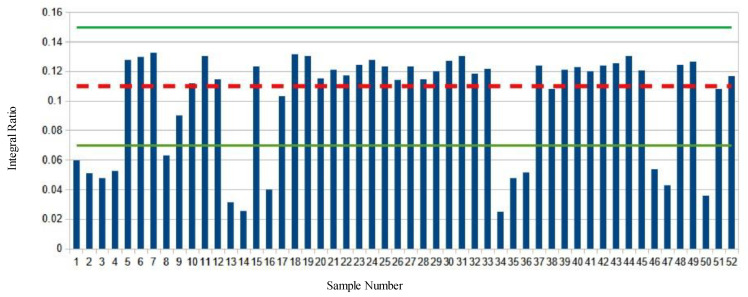
ω-3 Methyl to the remaining methyl protons ratio of market survey samples. The mean ratio of baseline samples is depicted with a dashed red line, green lines are the mean plus or minus two standard deviations.

**Figure 5:**
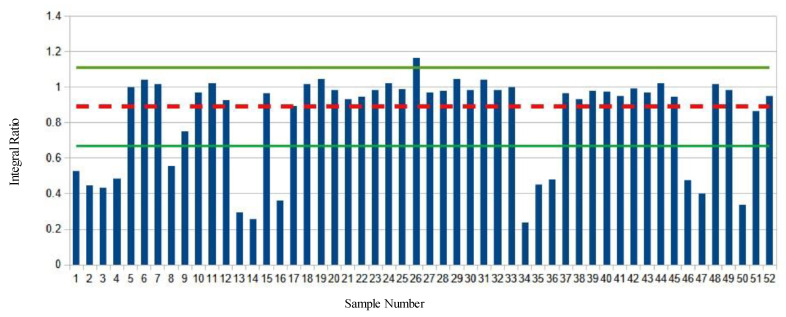
ω-3 Methyl protons to glycerol C2 proton ratio of the market survey samples. The mean ratio of baseline samples is depicted with a dashed red line, green lines are the mean plus or minus two standard deviations.

Analysis of the market survey cheese samples revealed many samples exhibiting significant deviations from both the values found in the baseline samples and the majority of the other survey samples tested. (Figure 2, Figure 3, Figure 4 and Figure 5, Supplementary Materials).

In Figure 2, the dashed red line is the average value of the unsaturated bonds to glycerol C2 proton ratio (A:B) of the baseline samples. The green lines represent the ± two standard deviations (S.D.) of the baseline sample ratio average. A number of samples (n = 17) appear to be outliers (deviation greater than two S.D.) based on the ratiometric analysis.

Figure 3 shows the polyunsaturated protons to glycerol C2 proton ratio (C:B) of the market survey samples. The average ratio of the baseline samples is shown with a red dashed line. The green lines indicate the mean plus or minus two S.D. Fourteen different samples show significant deviations from the average values.

Figure 4 shows the ω-3 methyl to remaining methyl proton ratio (D:E) of the market survey samples. The average ratio of the baseline samples is shown with a red dashed line. The green lines indicate the mean plus or minus two S.D. Again, 14 different samples show significant deviations from the average values, this time to lower integral ratios.

Figure 5 shows the ω-3 methyl protons to glycerol C2 proton ratio (D:B) of the market survey samples. The average ratio of the baseline samples is shown with a red dashed line. The green lines indicate the mean plus or minus two S.D. One sample shows a positive deviation as an outlier, while 14 samples have ratios significantly less than the baseline average.

Samples 1–4, 8, 9, 13, 14, 16, 34–36, 46, 47, and 50 all appear to be outliers, with ratiometric values falling outside of the 95% confidence interval or two standard deviations of the baseline sample mean. These samples consistently fall outside this interval in every ratiometric analysis and appear with different ratios compared to other market samples. Aside from sample 9, these consistently fall more than two standard deviations outside of the baseline mean. As such these samples were presumed to be adulterated and further investigation was pursued by comparing the spectra of one outlying sample to two presumably unadulterated samples. Due to failing two of the four ratiometric checks, sample 9 is also considered to be adulterated.

**Figure 6:**
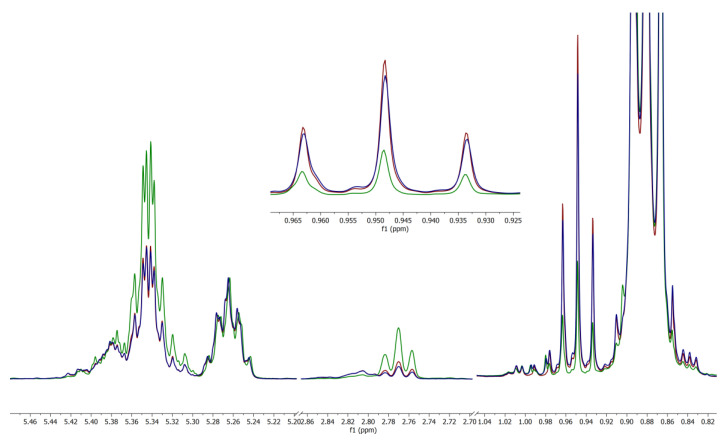
^1^H 500 MHz NMR spectral excerpt of samples 3 (green), 42 (blue), and B1 (maroon). Normalized for intensity to the glycerol peak at 5.265 ppm. Inset: Expansion of ω-3 terminal methyl region included for clarity.

Superimposed spectra of sample 3, sample 42, and baseline sample B1 show a radically different lipid profile in sample 3 versus the others when normalized for intensity to the 5.265 ppm glycerol C2 proton peak (Figure 6). The glycerol peak was used for normalization due to the majority of lipids in cheese being in the form of triacylglycerol [8]. The remaining adulterated samples showed overall similar deviation from the baseline spectra with varying degrees of deviation.

The ^1^H NMR spectra of samples 42 and B1 are very similar. However, sample 3 (green) deviates in the difference in intensity between the C2 glycerol proton peak at 5.265 ppm and the peak of the unsaturated bonds at 5.342 ppm. This is due to sample 3, relative to the C2 glycerol peaks, containing a higher abundance of unsaturated and polyunsaturated bonds than found in the other cheese samples. The ω-3 fatty acid levels are far lower than what would be expected of a genuine cheese sample. An overabundance of unsaturated bonds, polyunsaturated bonds, and low levels of ω-3 fatty acids in sample 3 would suggest adulteration of this sample with a vegetable-sourced oil. Despite being adulterated, sample 3 exhibited no remarkable olfactory or visual differences from any of the other grated cheese samples tested.

In order to identify the adulterant used, a series of intentionally adulterated samples were prepared using sample 19 as a model cheese. Samples were prepared with vegetable oil adulteration in ranges from 5–60 weight% and analyzed as described in the Methods Section. The adulterants used were Canola, grapeseed, peanut, olive, high oleic sunflower, high oleic safflower, high linolenic safflower, soybean, and palm oils. Most oils yielded lipid profiles inconsistent with the adulterated cheese samples, but palm oil yielded a lipid profile nearly identical to sample 3. The results are detailed in Table 2.

Comparing the other samples suspected of adulteration to intentionally adulterated cheese spectra shows that all of the suspicious samples appear to be adulterated with palm oil to varying degrees. A further study was conducted in order to more conveniently estimate the rate of palm oil adulteration in suspected samples using a calibration curve using integral ratios.

**Figure 7:**
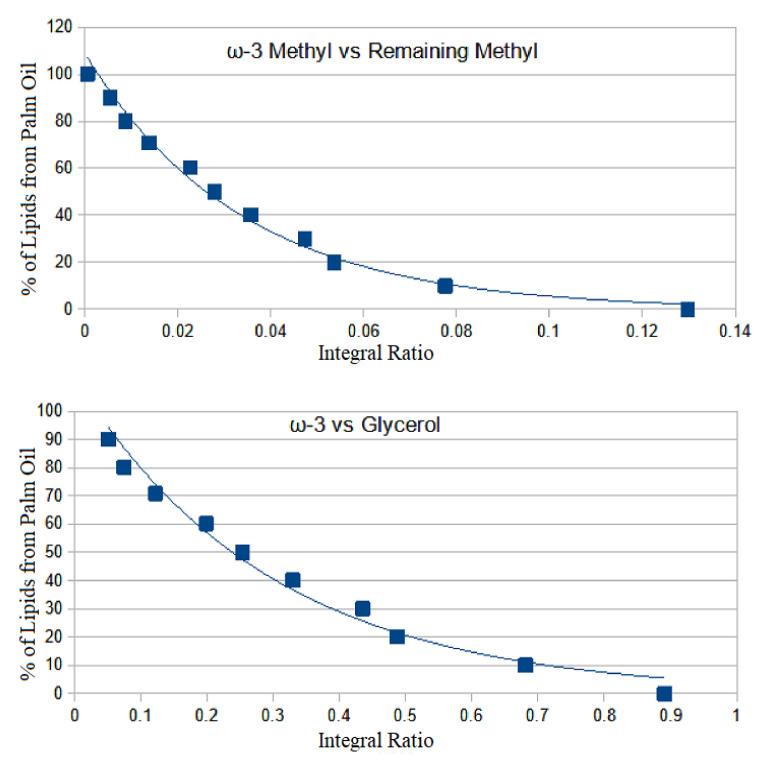
Calibration curves obtained from ^1^H NMR spectra of serially adulterated cheese using palm oil.

Quantification of the level of palm oil adulteration in these samples was accomplished by generating calibration curves of two peak ratios. This allows for a convenient and more precise estimation of the amount of palm oil in the sample versus the amount of actual cheese. It does not take into account the insoluble anti-caking agents present in all adulterated samples studied and, therefore, is not a measurement of the total *w*/*w* adulteration rate. Despite this limitation, the calculation does yield important information as to the degree of palm oil adulteration in these samples. Baseline sample B1 was used to make samples adulterated with palm oil in the range of 10 to 90 percent by weight. All samples were extracted and analyzed as described previously. The ratios of the intentionally adulterated cheese were calculated and plotted to generate a calibration curve allowing an estimation of the degree of palm oil adulteration versus cheese in each sample (Figure 7). The functions for each curve followed an exponential regression. The equation for ω-3 vs. remaining methyl peak was found to be F(x) = 108.9584 × 10^−29.7812^x. For the ω-3 vs. glycerol ratio, the equation was F(x) = −112.0973 × 10^−3.3871^x. The correlation coefficients for these curves are values of 0.989 and 0.983 respectively.

These calibration curves were used to calculate the approximate amounts of palm oil added to each sample relative to cheese with no accounting for any additional binders. The calculated values were found to be similar to the values determined by overlaying spectra of market survey samples with spectra of serially adulterated samples (Table 3). While this does not yield an exact determination of the level of adulteration, it does serve to calculate a general estimate of palm oil adulteration levels in these products.

## 3. Materials and Methods

### 3.1. Samples

Nine ungrated samples and one grated sample of various cheeses were analyzed to ascertain a lipid profile of unadulterated cheese samples. Of these baseline samples, three were Parmesan, two were Romano, and one was Asiago. To gain a broader understanding of cheese lipid profiles, one sample of Mimolette, one sample of Piave cheese, and one sample each of ungrated and pre-grated mozzarella were also analyzed.

All market survey grated hard cheese samples were obtained from retailers, restaurants, and public school cafeteria kitchens. All samples were composed of Parmesan, Romano, Asiago, and combinations thereof. All samples are detailed in Table 4.

Canola, grapeseed, peanut, olive, high oleic sunflower, high oleic safflower, high linolenic safflower, soybean, and palm oils were purchased from local and online retailers and used as received.

### 3.2. Sample Preparation

A 50 ± 2.5 mg sample of each cheese was placed in a 1.5 mL flip-top microcentrifuge tube. To this, 1 mL of deuterated chloroform (CDCl_3_ 99.8% D, 0.03% *v/v* TMS, Acros Organics, Switzerland) was added and the tube was agitated for 30 min. The resulting extract was removed via pipette and filtered through glass wool directly into a 5 mm NMR tube for analysis.

### 3.3. ^1^H NMR

The spectrometer used was a Bruker (Rheinstetten, Germany) Avance III HD spectrometer equipped with a TCI cryoprobe operating at 500.13 MHz. A proton experiment was performed with 16 scans (30° pulse, 20 ppm sweep width, 10 s relaxation time based upon 1.0 s T1, 65,536 data points, 300 K sample temperature). Spectra were processed and analyzed using Mestrenova 14.2 (Mestrelab, Santiago de Compostela, Spain). Processing parameters included referencing to TMS, 0.3 Hz apodization, exponential baseline correction, and automatic phase correction.

## 4. Conclusions

This study revealed a previously undiscovered method of adulterating pre-grated bovine hard cheeses for economic purposes. Palm oil itself is a clever adulterant owing to its semi-solid state at room temperature, similar color to cheese, and low price compared to cheese. Presumably, these adulterated products contain higher than normal levels of cellulose or other binders in order to maintain the appearance of the product. However, this study is strictly limited to the lipid profile of these products, and no attempts were made to quantify any fillers aside from palm oil.

In this study, 29% of all samples tested were certainly adulterated with palm oil. That combined with nearly half of the adulterated samples possessing lipid fractions composed of greater than 50% palm oil shows a rather brazen attitude in this industry regarding the commission of fraud through the adulteration of these products. The 52 samples tested are by no means an exhaustive survey of all pre-grated hard cheeses sold, however, it does reveal a new frontier in food adulteration. The method described herein will make the detection of this new type of food adulteration straightforward and aid in combating the problem.

## Figures and Tables

**Table 1:** Integrated regions used in this study.

Peak	Range	Chemical Shift (ppm)	Description	Integration Range (ppm)
A	Unsaturated Bonds	5.342	Unsaturated bond protons	5.44–5.30
B	Glycerol C2 Proton	5.263	C2 glycerol proton	5.29–5.23
C	Polyunsaturated	2.800	Methylene protons α to unsaturated bonds in polyunsaturated fatty acids	2.87–2.73
D	ω-3 Methyl	0.948	ω-3 fatty acid terminal methyl group	0.97–0.93
E	Non ω-3 Methyl	0.880	Terminal methyl group of all fatty acids aside from ω-3	0.91–0.85

**Table 2:** Adulterant identification trial results.

Adulterant Oil	Match?	Comments
Canola	No	ω-3 fatty acids too abundant
Grapeseed	No	ω-3 and polyunsaturated fatty acids too abundant
Peanut	No	ω-3, polyunsaturated and total unsaturated bonds too abundant
Olive	No	ω-3, polyunsaturated and total unsaturated bonds too abundant
Sunflower	No	ω-3, polyunsaturated and total unsaturated bonds too abundant
High Oleic Safflower	No	ω-3 fatty acids too abundant
High Linoleic Safflower	No	ω-3, polyunsaturated and total unsaturated bonds too abundant
Soybean	No	ω-3 fatty acids too abundant, insufficient levels of polyunsaturated fatty acids
Palm	Yes	near perfect match at 40% (*w*/*w*) adulteration

**Table 3:** Calculated levels of palm oil in adulterated samples.

Sample Number	Adulteration Level by Spectral Comparison	Calculated Adulteration Level
1	30%	31%
2	35%	46%
3	50%	49%
4	60%	54%
8	20%	21%
9	15%	11%
13	60%	59%
14	70%	73%
16	40%	35%
34	70%	65%
35	60%	51%
36	60%	55%
46	30%	26%
47	60%	54%
50	60%	56%

**Table 4:** List of all cheese samples analyzed in this study.

Sample	Variety	Form	Type of Sample
B1	Parmesan	Ungrated	Baseline
B2	Parmesan	Ungrated	Baseline
B3	Parmesan	Ungrated	Baseline
B4	Piave	Ungrated	Baseline
B5	Asiago	Ungrated	Baseline
B6	Romano	Ungrated	Baseline
B7	Romano	Ungrated	Baseline
B8	Mimolette	Ungrated	Baseline
B9	Mozzarella	Ungrated	Baseline
B10	Mozzarella	Ungrated	Baseline
1	Parmesan and Romano	Grated	Market Survey
2	Romano	Grated	Market Survey
3	Parmesan	Grated	Market Survey
4	Parmesan and Romano	Grated	Market Survey
5	Parmesan	Grated	Market Survey
6	Parmesan and Romano	Grated	Market Survey
7	Romano	Grated	Market Survey
8	Parmesan	Grated	Market Survey
9	Parmesan	Grated	Market Survey
10	Parmesan, Romano, and Asiago	Grated	Market Survey
11	Parmesan	Grated	Market Survey
12	Parmesan and Romano	Grated	Market Survey
13	Parmesan	Grated	Market Survey
14	Romano	Grated	Market Survey
15	Romano	Grated	Market Survey
16	Parmesan	Grated	Market Survey
17	Parmesan	Grated	Market Survey
18	Parmesan	Grated	Market Survey
19	Parmesan	Grated	Market Survey
20	Parmesan and Romano	Grated	Market Survey
21	Parmesan	Grated	Market Survey
22	Parmesan and Romano	Grated	Market Survey
23	Parmesan	Grated	Market Survey
24	Parmesan	Grated	Market Survey
25	Parmesan	Grated	Market Survey
26	Parmesan	Grated	Market Survey
27	Parmesan	Grated	Market Survey
28	Parmesan	Grated	Market Survey
29	Parmesan, Romano, and Asiago	Grated	Market Survey
30	Parmesan	Grated	Market Survey
31	Parmesan	Grated	Market Survey
32	Parmesan	Grated	Market Survey
33	Parmesan and Romano	Grated	Market Survey
34	Parmesan	Grated	Market Survey
35	Parmesan	Grated	Market Survey
36	Parmesan	Grated	Market Survey
37	Parmesan	Grated	Market Survey
38	Parmesan	Grated	Market Survey
39	Parmesan	Grated	Market Survey
40	Parmesan	Grated	Market Survey
41	Parmesan and Romano	Grated	Market Survey
42	Parmesan	Grated	Market Survey
43	Parmesan	Grated	Market Survey
44	Parmesan	Grated	Market Survey
45	Parmesan	Grated	Market Survey
46	Parmesan	Grated	Market Survey
47	Parmesan and Romano	Grated	Market Survey
48	Parmesan, Romano, and Asiago	Grated	Market Survey
49	Parmesan	Grated	Market Survey
50	Parmesan	Grated	Market Survey
51	Parmesan	Grated	Market Survey
52	Parmesan	Grated	Market Survey

## Data Availability

Original data are available upon request.

## Supplementary Materials

The following supporting information can be downloaded at: https://www.mdpi.com/article/10.3390/molecules28030920/s1, Table S1: Raw integral values of cheese spectra for all samples; Table S2: Cheese integral ratios for all samples.

## Sample Availability

Samples of the cheeses are available from the authors.
