# Supplementary material for: Detection of Vegetable Oil Adulteration in Pre-Grated Bovine Hard Cheeses via 1H NMR Spectroscopy
Source: Molecules. 2023 Jan 17;28(3):920. doi: 10.3390/molecules28030920 (PMC9919626; doi:10.3390/molecules28030920)
Supplement: Supplementary file 1 [file molecules-28-00920-s001.zip › molecules-2156176-supplementary.pdf]

Detection of Vegetable Oil Adulteration in Pre-Grated Bovine Hard Cheeses Via ^{1}H NMR Spectroscopy

Colleen L. Ray [1], James A. Gawenis [2], Madison P. Bylo [1], Jonny Pescaglia [1], and C. Michael Greenlief [1,*]

Supplemental material:

Table S1. Raw integral values of cheese spectra for all samples.

Sample	Unsaturated Bond Raw Integral	Triglyceride C2 Raw Integral	Polyunsaturated Raw Integral	ω3 Methyl Raw Integral
**Integration Range (ppm)**	5.44–5.30	5.29–5.23	2.87–2.73	0.97–0.93
**B1**	11215.03	6122.93	1507.62	5867.57
**B2**	13330.51	7326.82	1786.93	6523.03
**B3**	16721.61	9873.71	2058.03	9654.26
**B4**	19120.96	9915.66	2398.15	9495.54
**B5**	12633.80	7511.8	1750.22	6795.81
**B6**	13392.30	7256.98	1905.7	5485.73
**B7**	14440.66	7160.02	2057.87	4612.56
**B8**	16858.04	11285.08	1703.53	10900
**B9**	13560.41	7564.52	1763.59	7293.11
**B10**	9598.85	5555.48	1296.17	5260.06
**1**	29517.02	10284.10	4468.45	5419.31
**2**	30490.16	10523.53	4598.03	4720.94
**3**	29149.25	9828.02	4397.71	4245.48
**4**	28481.36	9292.80	4323.40	4499.07
**5**	23567.49	13155.93	2805.12	13134.30
**6**	22827.78	12886.91	2796.74	13459.23
**7**	21543.35	11747.87	3217.43	11939.72
**8**	24574.88	9402.02	3567.79	5221.23
**9**	24233.60	10812.65	3364.62	8115.01
**10**	15873.56	7846.85	1968.87	7599.99
**11**	26441.74	14705.49	3273.75	15018.02
**12**	25361.17	14291.36	3117.73	13290.77
**13**	18807.29	5548.86	2979.17	1633.16
**14**	15386.89	4082.96	2520.28	1046.20

**15**	22006.39	12258.78	2776.24	11842.54
**16**	29979.40	9661.08	4620.69	3492.43
**17**	13671.69	6961.02	1752.13	6241.94
**18**	16402.28	9205.36	2022.72	9377.52
**19**	15234.79	8064.80	2053.37	8458.72
**20**	15251.12	6933.54	1977.17	6825.74
**21**	15564.70	9201.76	1921.62	8598.86
**22**	16954.23	9608.00	2033.48	9109.76
**23**	15673.78	8661.35	2001.03	8532.18
**24**	17217.09	9404.12	2100.22	9616.26
**25**	14541.49	8166.92	1812.29	8074.21
**26**	13631.51	6010.32	1648.23	7004.57
**27**	14411.33	8542.10	1748.98	8273.64
**28**	10240.96	5116.26	1283.58	5018.73
**29**	12819.18	6649.26	1532.18	6965.63
**30**	14267.80	7727.85	2160.02	7600.48
**31**	12923.47	7242.64	1549.16	7551.71
**32**	12341.41	6826.59	1553.88	6724.32
**33**	14226.59	7920.62	1664.14	7922.23
**34**	24518.64	7565.97	4210.70	1785.55
**35**	18988.60	5791.81	2998.43	2618.79
**36**	14749.27	4914.61	2153.04	2365.61
**37**	11311.17	6455.03	1443.40	6220.89
**38**	12099.86	6117.10	1391.19	5693.14
**39**	11511.96	6481.73	1431.11	6355.87
**40**	12611.94	7187.84	1534.14	7002.98
**41**	12269.11	6993.78	1507.41	6672.01
**42**	10373.24	5828.77	1193.18	5789.26
**43**	12536.51	7127.68	1633.60	6911.82
**44**	18986.99	11212.00	2315.47	11496.46
**45**	18917.02	10793.04	2426.96	10240.92
**46**	23901.96	8434.01	3668.47	4011.97
**47**	26271.81	8174.04	4088.53	3249.94
**48**	23490.27	13230.16	2907.87	13495.98
**49**	22411.74	13121.77	2813.55	12945.46
**50**	29046.62	8785.84	4619.22	2952.94
**51**	12267.37	7569.59	1714.65	6550.53
**52**	13776.43	7627.66	1790.21	7244.01

Table S2. Cheese integral ratios for all samples.

Sample	Unsaturated Bonds vs. Glycerol C2 Ratio	Polyunsaturated vs. Glycerol C2 Ratio	ω3 Methyl vs. Remaining Methyl Ratio	ω3 Methyl vs. Glycerol C2 Ratio
**B1**	1.831644	0.246225	0.117850	0.958294
**B2**	1.819413	0.243889	0.129701	0.890295
**B3**	1.693549	0.208435	0.123275	0.977774
**B4**	1.928360	0.241855	0.117452	0.957631
**B5**	1.681861	0.232996	0.107207	0.904685
**B6**	1.845437	0.262602	0.081174	0.755925
**B7**	2.016846	0.287411	0.067784	0.644210
**B8**	1.493834	0.150954	0.119308	0.965877
**B9**	1.792633	0.233140	0.121001	0.964121
**B10**	1.727816	0.233314	0.121384	0.946824
**1**	2.870161	0.434501	0.059583	0.526960
**2**	2.897332	0.436928	0.050980	0.448608
**3**	2.965933	0.447467	0.048042	0.431977
**4**	3.064885	0.465242	0.052595	0.484146
**5**	1.791397	0.213221	0.127508	0.998356
**6**	1.771393	0.217022	0.129916	1.044411
**7**	1.833809	0.273873	0.132512	1.016331
**8**	2.613787	0.379471	0.063308	0.555331
**9**	2.241227	0.311174	0.090138	0.750511
**10**	2.022921	0.250912	0.111818	0.968540
**11**	1.798086	0.222621	0.130623	1.021253
**12**	1.774581	0.218155	0.114691	0.929986
**13**	3.389397	0.536898	0.031562	0.294324
**14**	3.768563	0.617268	0.025438	0.256236
**15**	1.795153	0.226470	0.123481	0.966046
**16**	3.103111	0.478279	0.040141	0.361495
**17**	1.964035	0.251706	0.103302	0.896699
**18**	1.781818	0.219733	0.131397	1.018702
**19**	1.889047	0.254609	0.130511	1.048844
**20**	2.199615	0.285160	0.115302	0.984452
**21**	1.691492	0.208832	0.121023	0.934480
**22**	1.764595	0.211644	0.117282	0.948143
**23**	1.809623	0.231030	0.124763	0.985087

**24**	1.830803	0.223330	0.127658	1.022558
**25**	1.780535	0.221906	0.123193	0.988648
**26**	2.268017	0.274233	0.114241	1.165424
**27**	1.687095	0.204748	0.123297	0.968572
**28**	2.001650	0.250882	0.114530	0.980937
**29**	1.927911	0.230429	0.120420	1.047580
**30**	1.846283	0.279511	0.127309	0.983518
**31**	1.784359	0.213894	0.130280	1.042674
**32**	1.807844	0.227622	0.118449	0.985019
**33**	1.796146	0.210102	0.121922	1.000203
**34**	3.240647	0.556531	0.025259	0.235997
**35**	3.278526	0.517702	0.047650	0.452154
**36**	3.001107	0.438090	0.051898	0.481342
**37**	1.752303	0.223609	0.124178	0.963728
**38**	1.978039	0.227426	0.107985	0.930693
**39**	1.776063	0.220791	0.121196	0.980582
**40**	1.754622	0.213435	0.122717	0.974282
**41**	1.754289	0.215536	0.120147	0.953992
**42**	1.779662	0.204705	0.123947	0.993222
**43**	1.758849	0.229191	0.125583	0.969715
**44**	1.693453	0.206517	0.130724	1.025371
**45**	1.752705	0.224863	0.120957	0.948845
**46**	2.833997	0.434962	0.054101	0.475690
**47**	3.214054	0.500185	0.042771	0.397593
**48**	1.775509	0.219791	0.124449	1.020092
**49**	1.707981	0.214418	0.126485	0.986564
**50**	3.306072	0.525757	0.035746	0.336102
**51**	1.620612	0.226518	0.107960	0.865374
**52**	1.806115	0.234700	0.117029	0.949703
